# Supplementary material for: Amplification of Hippo Signaling Pathway Genes Is Governed and Implicated in the Serous Subtype-Specific Ovarian Carcino-Genesis
Source: Cancers (Basel). 2024 May 5;16(9):1781. doi: 10.3390/cancers16091781 (PMC11082992; doi:10.3390/cancers16091781)
Supplement: Supplementary file 1 [file cancers-16-01781-s001.zip › Supplementary figures.pdf]

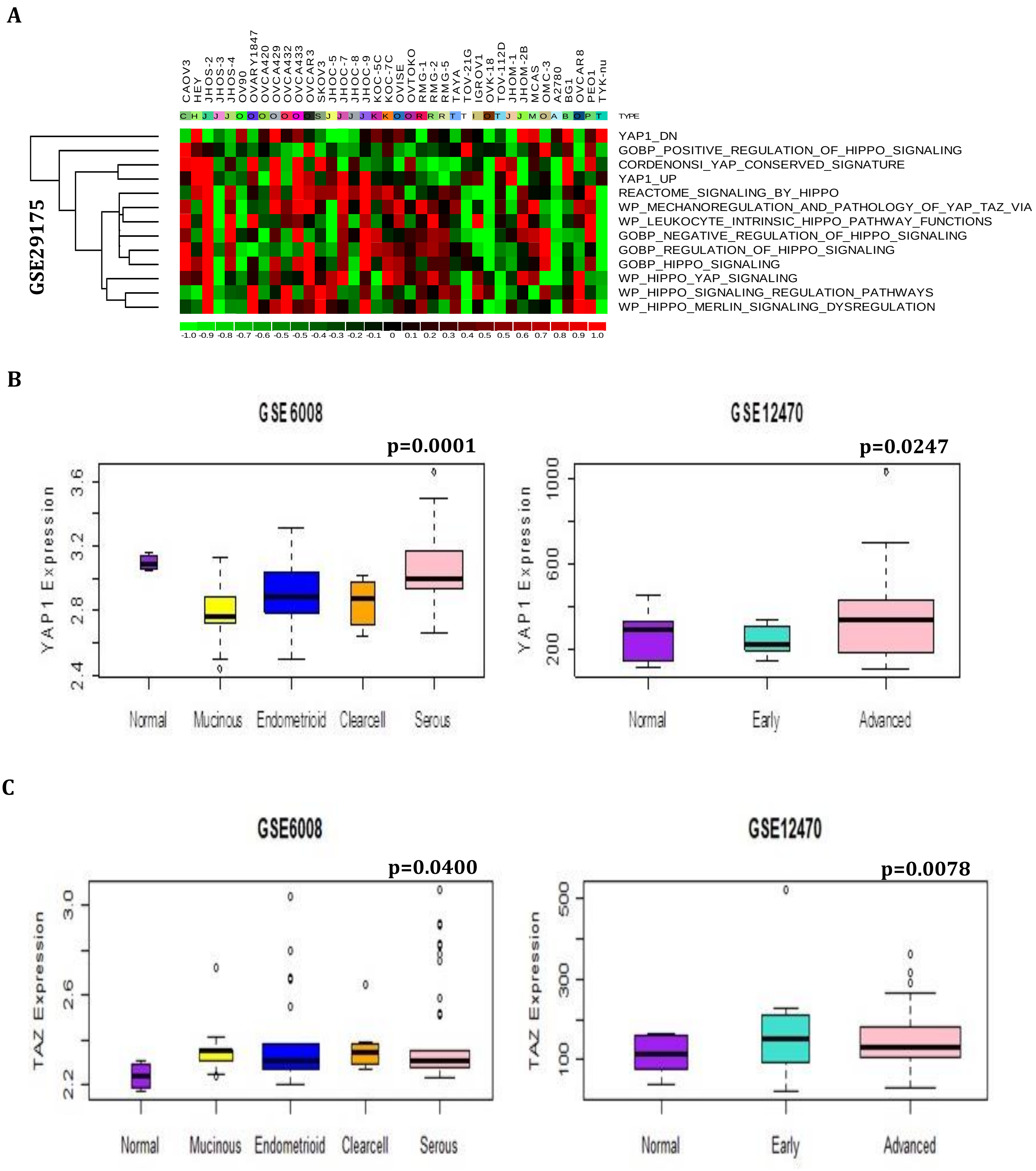

Figure. S1 The impact of these signaling pathway signatures was further validated with ovarian cancer cell-line profiles GSE29175. **A**) The activation pattern indicates that Hippo signatures are elevated significantly across the cell lines of ovarian tumors. Moreover, the individual gene expression of Hippo pathway downstream genes YAP1 and TAZ were further ratified in the profiles GSE6008 and GSE12470. **B-C**) The boxplot results revealed that YAP1 and TAZ genes are significantly expressed in serous or advanced serous subtype ovarian tumors. A one-way ANOVA test measured the significant p values ($p<0.05$).
